# Migrating Giant Honey Bees (*Apis dorsata*) Congregate Annually at Stopover Site in Thailand

**DOI:** 10.1371/journal.pone.0044976

**Published:** 2012-09-19

**Authors:** Willard S. Robinson

**Affiliations:** Biology Department, Casper College, Casper, Wyoming, United States of America; University of Arizona, United States of America

## Abstract

Giant honey bees (*Apis dorsata*) of southern Asia are vital honey producers and pollinators of cultivated crops and wild plants. They are known to migrate seasonally up to 200 km. It has been assumed their migrations occur stepwise, with stops for rest and foraging, but bivouacking bees have rarely been seen by scientists. Here I report discovery of a site in northern Thailand where bivouacs appeared in large congregations during the wet seasons of 2009 and 2010. The bivouac congregation stopover site is a small mango orchard along the Pai River. Bivouacs rested in branches of mango and other tree species in the immediate vicinity. Departures were preceded by dances indicating approximate direction and apparently, distance of flights. Such consistent stopover sites likely occur throughout southern Asia and may support critical, vulnerable stages in the life history of giant honey bees that must be conserved for populations of the species to survive.

## Introduction

The giant honey bee, *Apis dorsata,* is an important pollinator [1], [2] and honey producer [3] that ranges through southern Asia from Pakistan to Indonesia [4]. Colonies migrate seasonally. Though details vary with region, colonies typically live in lofty communal nest sites of ≈20–100 colonies at relatively high elevation (>1,000 m) during the dry season. There they build ≈1.5-m-diameter single wax combs. The colonies grow quickly and reproduce by fission (swarming). As forage decreases toward the end of the season, colonies abandon their combs and migrate to lower elevations, establishing new nests there for the mass flowering of the monsoon season [5]–[9]. Microsatellite DNA fingerprinting studies indicate that returning colonies are faithful to original nesting sites after many months or even years away [10], [11].

Migration distances have been inferred by the presence of colonies on combs in different places at different times of year. Swarms reportedly cross the 50-km-wide Strait of Malacca between Sumatra and Malaysia [12]. Colonies in Sri Lanka travel for a month or so, up to 200 km in each direction, resting in trees along the way [13]. At stopovers the bees form combless clusters, or bivouacs, and accumulate food reserves for flight and for comb construction upon arrival at nest sites [7], [13], [14]. Shorter, altitudinal migrations of the closely related montane giant honey bee *Apis laboriosa* are well documented [15]–[17]. Widely dispersed bivouacs of this species survive the coldest months of Himalayan winter huddling near the ground, “…no swarm…visible from the site of another” [15].

Bivouacking *A. dorsata* have received no systematic study. Rare, opportunistic discoveries of bivouacs allowed researchers to describe dances of scout bees and subsequent flights of widely scattered Sri Lankan swarms, which were assumed to be near the end of migrations and seeking nearby nest sites [13].

Although I recently described congregated bivouacs [18], no one has reported annual congregations at traditional sites. Here I report timing and phenology of arrival and departure of 52 *A. dorsata* bivouacs found over 2 consecutive monsoon seasons, congregated in and around a riverside mango grove in northern Thailand. As the same site was occupied in consecutive years, and knowledgeable field workers report that groups of giant honey bees gather there annually, I call it a “bivouac congregation stopover site” (BCSS). I describe the aggregated nature of the bivouacs, some of their general features, dances the bees performed in preparation for departure flights, and ensuing flight directions.

Finally I stress the possible implications of BCSS’s for conservation of this spectacular honey bee, in light of worldwide pollinator decline, Southeast Asia’s rapid deforestation, dwindling numbers of giant honey bees and other insect species’ vulnerability to ecological bottlenecks.

## Study Site

### Timing of Bivouac Searches

I began searching for bees on 25 August 2009 at the Mae Hong Son Agricultural Research and Development Center. The Center is 4 km southwest of Mae Hong Son, Thailand, elevation 200 m, 19°16′09 N, 97°56′39 E. Searches were approximately weekly until 7 September, when I encountered 2 *A. dorsata* bivouacs in mango (*Mangifera indica*) trees in an orchard at the Center. Other swarms soon began to arrive at the study site. That season’s work concluded 31 October, when the last of 16 swarms departed. Deciduous trees were then shedding their leaves as the dry season commenced.

In 2010 I began searching for bivouacs at the Agricultural Center 17 August. Observations concluded 19 November, thus bracketing the 2009 study period. Although the study ended 3 weeks later than in the previous year, the dry season was not nearly as advanced.

### Physical Setting of Study Site

**Figure 1:**
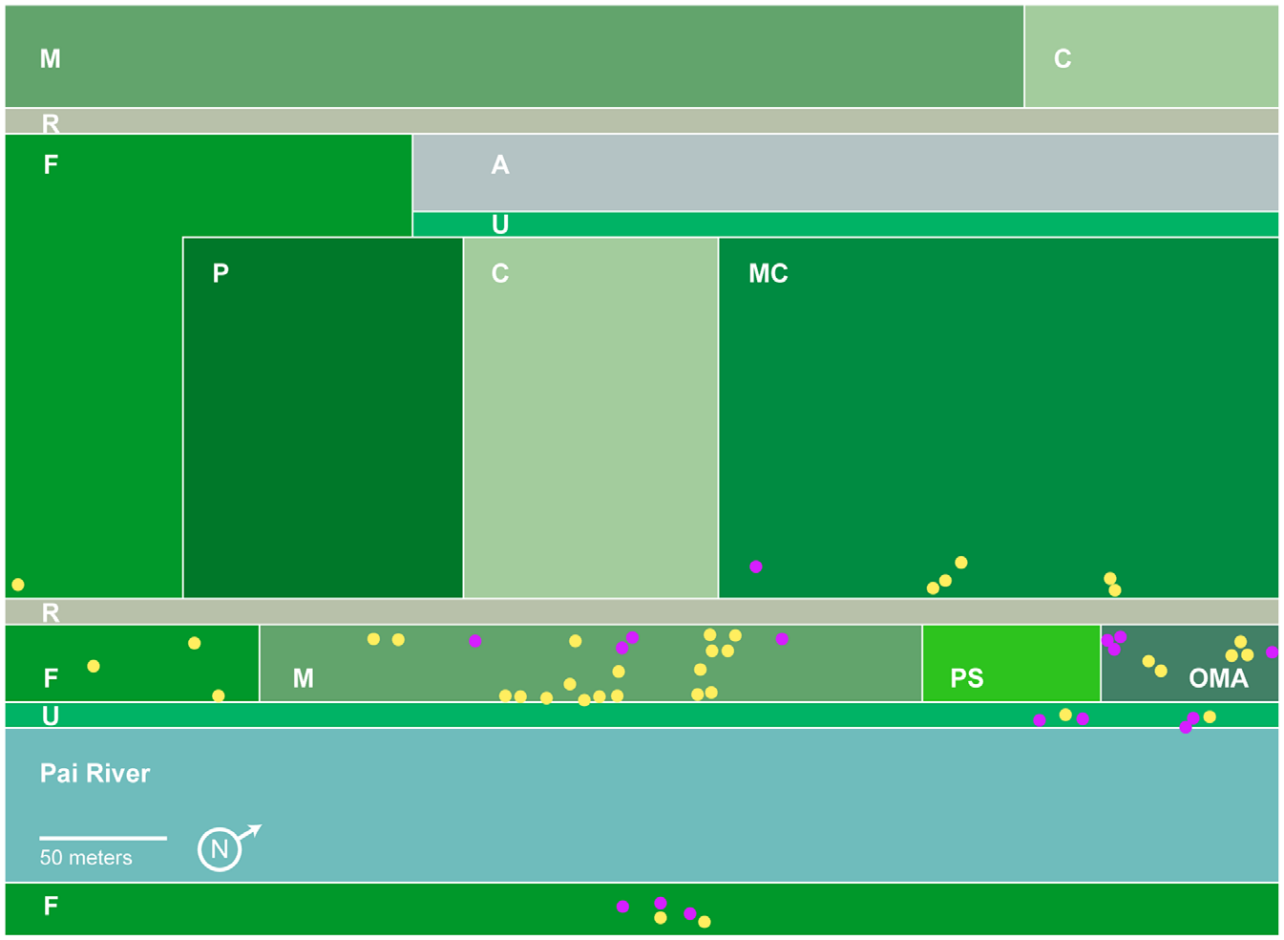
Study site map to scale, with bivouac locations marked for 2009 (purple dots) and 2010 (yellow dots). Note concentration of bivouacs in riverside mango trees. In 2010 one other bivouac was seen, 1 km NE of study site, along transect road. A  =  airstrip; C  =  assorted vegetable crops; F  =  forest; M  =  mango orchards; Ma  =  macadamia orchard; OMA  =  old mango trees; P  =  pomelo orchard; PS  =  passion fruit; R  =  road; U  =  undergrowth.

Fig. 1 depicts the mango orchard, its surroundings and bivouac locations for the two study years. The orchard is narrow, 4–8 trees in width, stretching north-south along the Pai River. In 2009, it was ≈ 350 m by ≈30 m. Tree removal reduced its length in 2010 to ≈270 m. It is bordered to the west by a narrow road partly lined by *Eucalyptus* and then a 150-m-wide strip comprising macadamia (*Macadamia integrifolia*) and pomelo (*Citrus maxima*) orchards, and mixed vegetable plantings. ≈250 m west of the river is another large mango grove. Directly east of the mango orchard is the Pai River, ≈70 m wide, with trees and undergrowth of various species along its bank. Dense moist broadleaf forest lies to the south; to the north is Research Center land cultivated with such plants as macadamia, scattered mango, vegetables, rice and ornamental flowers. Surrounding the Center on all sides, including the east side of the river, is steeply rising, relatively undisturbed mountain forest of diverse deciduous species.

**Figure 2:**
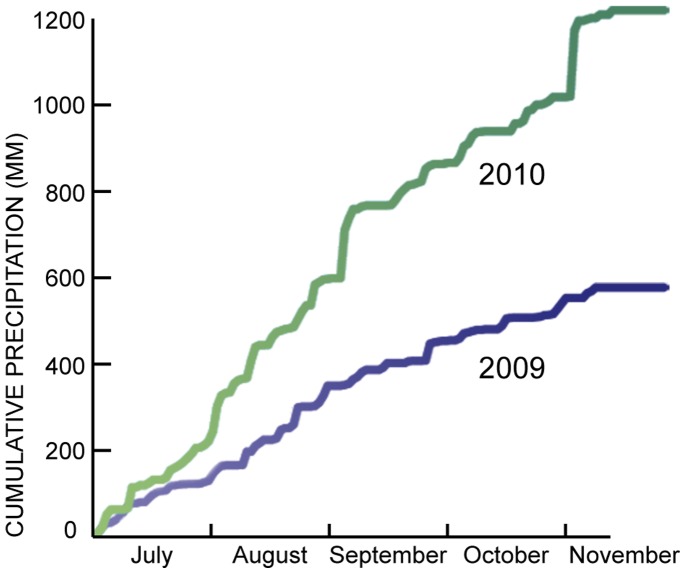
Cumulative rainfall, Mae Hong Son, July-November 2009 and 2010. Source: Air Force Datsav3 Weather Station 483000, call sign VTCH.

The rainy season in northern Thailand is April through September, peaking in August. The dry season is November to February. The summer and fall of 2009 were abnormally dry, while in 2010 a strong monsoon caused widespread flooding. From July through October, more than twice the amount of rain fell in 2010 as in 2009 (Fig. 2).

## Results

### Features of Bivouacs

**Figure 3:**
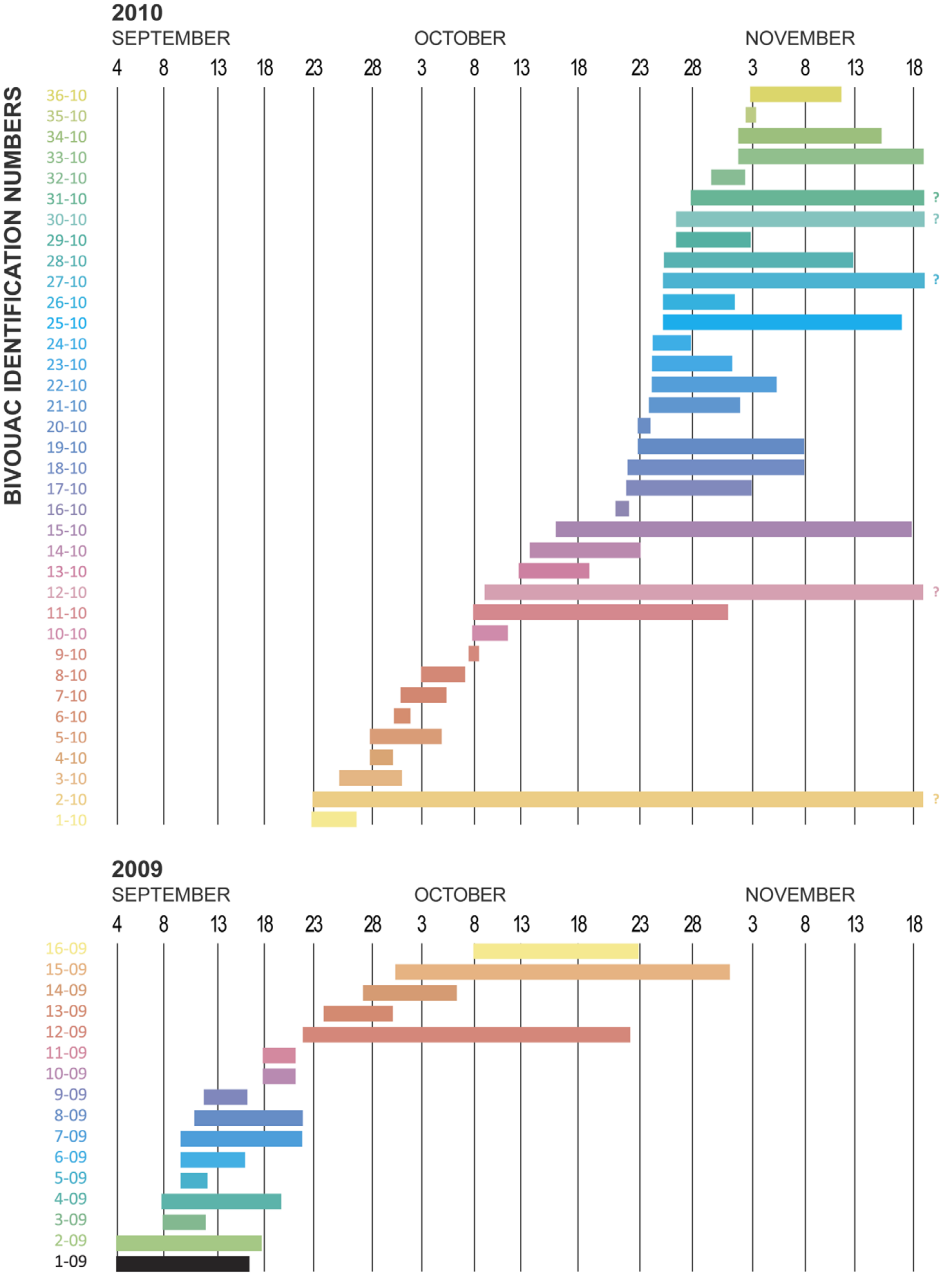
Timeline chart of bivouac arrival and departure, 2009 and 2010. Bars followed by a question mark represent bivouacs still on site at conclusion of 2010 study.

Dates of arrival and departure of bivouacs are presented in Fig. 3. Arrival of bivouacs was later and more protracted in the wet autumn of 2010 than in the dry 2009. In 2009, bivouacs remained in the study area for 11.1± s.d. 8.8 days; 2010 mean stay was 12.5± s.d. 12.1 days. The difference of 1.4 days was not significant according to an unpaired t-test (2-tailed P = 0.68; df = 50). Median duration of residence for 2009 and 2010 was 11 and 8.5 days respectively. Stays ranged from 2–31 days in 2009; 2 hours to ≥57 days in 2010. Concentration of bivouacs in the core area of the study site was such that I frequently saw and filmed swarms landing nearby as I studied the bivouacs; at one point in 2010, 12 bivouacs were simultaneously crowded within an area of 2,100 m^2^, all on mango. If not for the tree foliage all would have been easily visible from the center of the occupied area.

Bivouacs favored mango trees more in 2010 and were also more densely concentrated than in 2009 (Fig. 1). Bivouacs all hung from tree branches, comprising 10 tree species. Of the 52 total bivouacs, 28 rested in mango, 7 in *Acacia* sp., 6 in macadamia, with lesser numbers in other species. In 2009, 6 of 16 were in *Acacia*, 5 in mango, 5 others distributed among several species. In 2010, 23 of the 36 bivouacs rested in mango, 5 in macadamia, with the 8 others distributed among various species. Miscellaneous trees utilized included native species of teak (*Tectona grandis)*, cluster fig (*Ficus racemosa*), *Dipterocarpus* sp. and 4 unidentified species.

**Figure 4:**
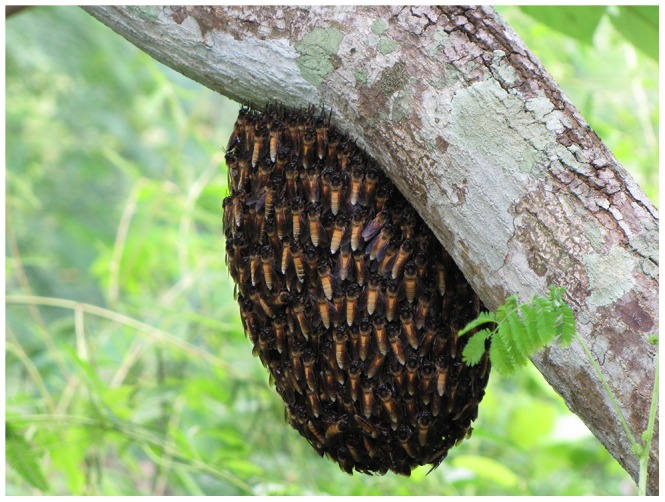
The smallest bivouac at the study site.

**Figure 5:**
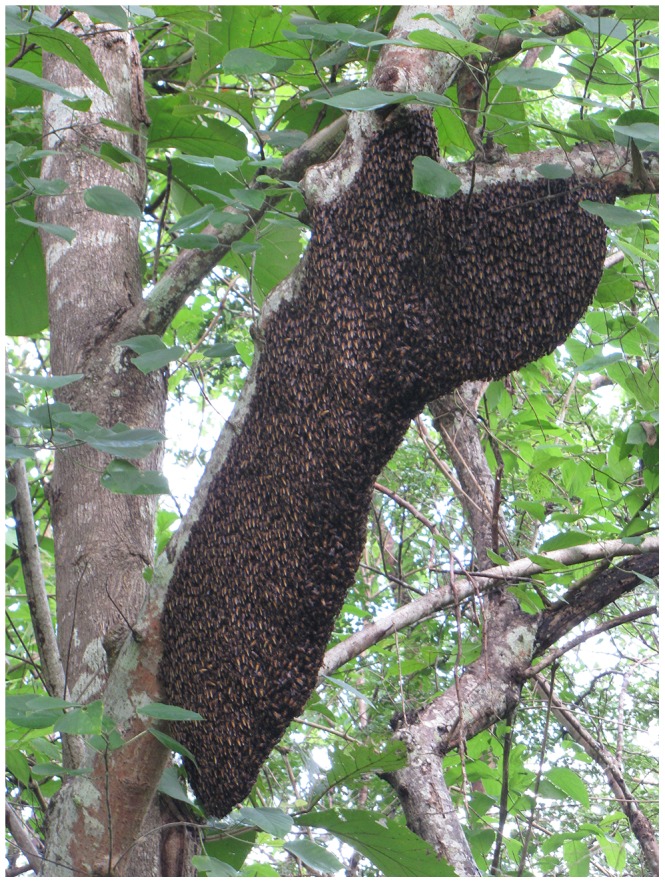
One of the largest bivouacs at the study site.

Mean size of 2009 bivouacs was 1,807± s.d. 683 cm^2^; in 2010 mean size was 2,562± s.d. 3,301 cm^2^. This difference was not significant according to an unpaired t-test (2-tailed P = 0.37; df = 50). However, size range and variability were markedly different in the 2 years, as reflected in disparate coefficients of variation. In 2009 bivouacs ranged in size from 226 to 2,743 cm^2^; in 2010 the range was 130 to 12,993 cm^2^. The coefficient of variation [19] in 2010 was 3.4 times that of 2009. Six of the 2010 bivouacs were smaller than 500 cm^2^ and 3 were larger than 11,000 cm^2^ (Figs. 4, 5). Rough estimates of the numbers of individual bees, calculated from photographs, indicated that the smallest bivouacs contained a few hundred bees, the largest well over 100,000 bees.

Bivouacs generally rested at lower heights in 2010 than in 2009. Resting height varied from 1.8 to 18.0 m (mean  = 10.1±5.0 m) in 2009; in 2010 resting height ranged from 1.0 to 15.0 m (mean  = 5.6±4.3 m). This 4.5-m difference in mean resting height was statistically significant (2-tailed P = 0.002; df = 50).

None of the 52 bivouacs built comb. Departing swarms left numerous small beads of white wax behind on the bark at their resting places. In 2010, 2 transitory colonies, not included among the bivouacs in this study, established small (≈30 cm-diameter) combs near the study site during the first week, only to abscond within a few days.

### Departure Dances and Flights

All swarm flights were preceded in the 30–60 min before liftoff by a varying number of simultaneously dancing bees, spread over the outer curtain of bees (Video S1, Video S2). Number of dancers increased with bivouac size. Dancers indicated unanimously (r = 1, Rayleigh test) [20] and fairly precisely the direction, and apparently the relative distance of the upcoming flight. For longer dances (waggle runs >3 sec) these were distinctive, staggering runs [13], [18], [21]. I describe details of these dances elsewhere (W.S. Robinson, in preparation).

**Figure 6:**
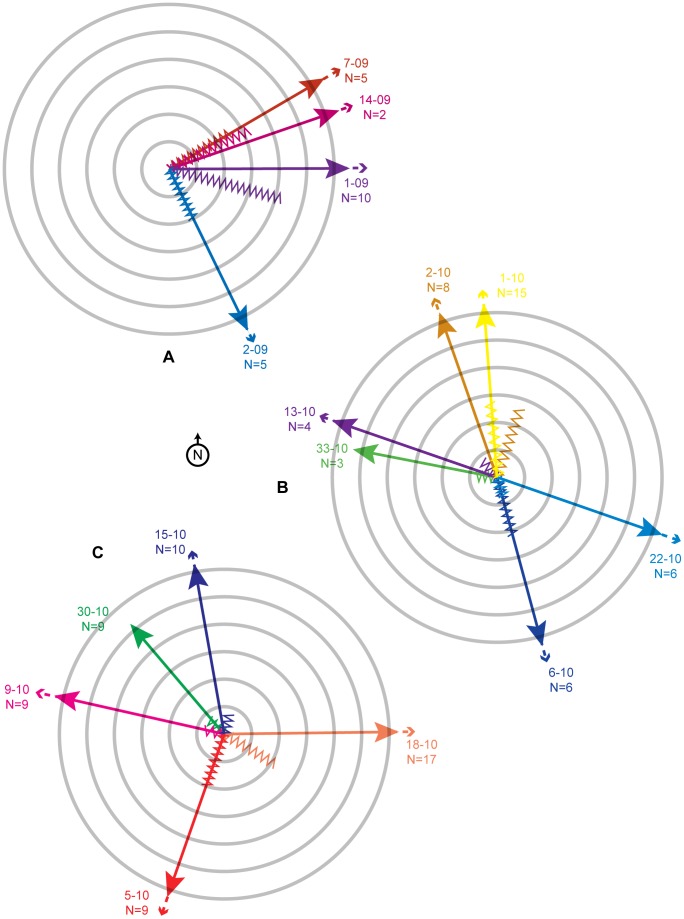
Compass plots depicting flight directions indicated by dancers and following flights. (A) observations from 2009, (B, C) observations from 2010. Dances of each worker were numerous; directions and distances indicated were unanimous for all dancers in each bivouac. Each color represents dances and flight of a single bivouac. Each concentric ring represents either 3 s of dancing (zigzag lines)–waggle runs only for dances of >3 s duration, complete circuits for dances of <3 s–or 30 m of flight (straight lines). Most flights exceeded 150 m and disappeared from view, as indicated by broken lines. At periphery are identification numbers of bivouacs. N  =  maximum number of workers simultaneously dancing within 30 min before flight.

I watched 4 swarms depart in 2009 (Video S3). In each, dancers indicated impending flights ranging from ENE to SE, and flights were generally easterly (Fig. 6A). I thus hypothesized [18] that all bivouacs might be *en route* to a known annual nesting aggregation near the village of Pai, about 50 km E. However, in 2010 the dances and flights of 11 witnessed departures were oriented to widely varying compass directions (Fig. 6B, C). I traced the flights of 2 of these bivouacs that took flights of ≈150 m, both preceded by dances with waggle circuits of 2 s (Fig.6b, 6c). Both bivouacs made these short flights in the afternoon, settled, and had departed the study site unobserved by the next morning. All other flights were impossible to trace, mostly disappearing from view after ≈ 150–200 m.

Flight directions of the departing bivouacs quite accurately reflected preceding waggle dance orientations (Fig. 6). For each of the 15 witnessed flights, the probability that the direction indicated by unanimously dancing bees would by chance alone be so close to the departure direction is extremely low (p<0.001, Rayleigh V-test) [20].

## Discussion

### Functions of Stopover Site

**Figure 7:**
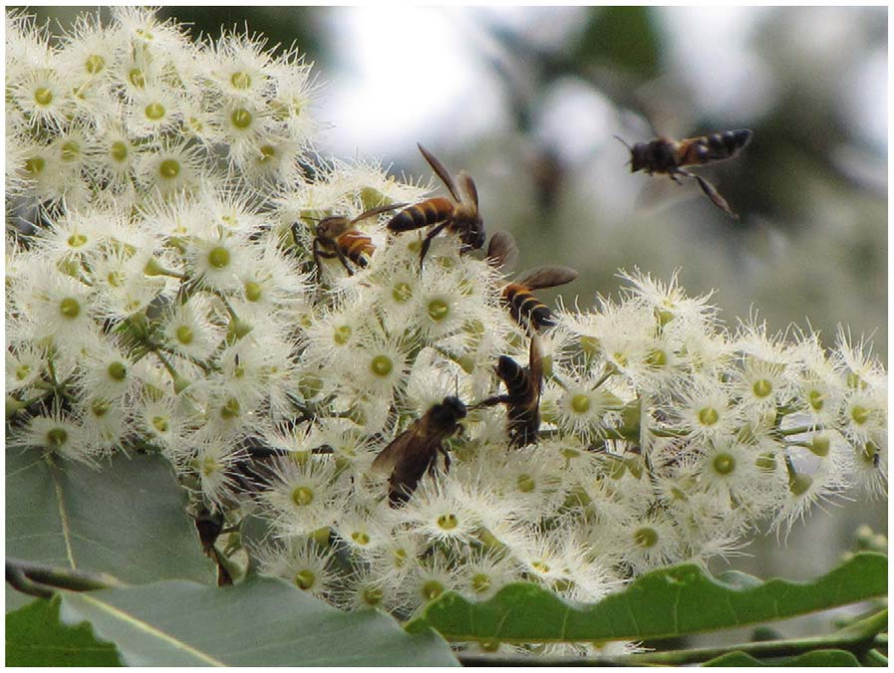
*A. dorsata* folragers in high density on *Eucalyptus* flowers.

It is possible that BCSS’s occur as migration stopover grounds throughout southern Asia, likely in geographical locations with landmarks that are easily recognizable to bees and could be found instinctively, just as drone congregation areas are found by both instinct and environmental cues [22]. This is especially likely considering evidence that *A. dorsata* migration has a genetic basis [13]. At this BCSS, the Pai River is a pronounced navigational landmark and a water source to traveling bees. Flowering native teak and assorted understory herbs provide nectar, as do plentiful non-native *Eucalyptus* trees (Fig. 7). In addition, numerous *A. cerana* swarms utilize the site as a refuge in defense against hornet attacks (W. S. Robinson, submitted for publication).

### Migration Origin and Destination Questions

Without marking or radio telemetry studies, it is impossible to know either the origin(s) or ultimate destination(s) of the migrating bees. Given the variety of flight directions from this valley surrounded by higher ground, it appears the bees are making altitude shifts of varying distance similar to what occurs in Nepal [15], but related more to a search for forage as the dry season commences [7], [14]. This would explain the later colony arrivals and departures in the intense, prolonged 2010 wet season.

An alternative hypothesis to account for the BCSS is that bees were considering, but discarding, the mango orchard as a possible nesting site. This alternative is supported by the presence in 2010 of 2 small transitory colonies that built comb and quickly absconded, but seems unlikely in that 52 swarms built no comb.

### Bivouac Features

The 2010 bivouacs, larger in number, were also more densely crowded than in 2009. Unlike those in Sri Lanka [13], bivouacs did not generally rest in exposed branches above surrounding vegetation. Many rested low in shady, protected places. This may be a function of the mango trees themselves, most of which were pruned to <5 m in height. The greater concentration in the mangoes in 2010 thus also accounts for those bivouacs resting at significantly lower heights than those of 2009.

The range in size of the bivouacs was remarkable, given the typical *A. dorsata* colony population of 35,000–40,000 bees [8]. The smallest, 130 cm^2^ surface area on one side, (approximately equal to a circle with a diameter of 13 cm) was about the size of a grapefruit and contained a few hundred bees; the 3 largest, >11,000 cm^2^ (approximately equal to a circle with diameter 118 cm), were as large as average-sized adult humans and comprised >100,000 bees. Yet all functioned normally, with foragers arriving and departing, ventilators fanning in the heat, defensive shimmering to deter hornets, frequent mass defecation flights, normal dancing and eventual flight. Also notable was the range in duration of stay (2 hr to >57 days). Assuming simply that the bees are resting and foraging until they have adequate food or fat reserves in their bodies to fuel further flight does not seem to account for this huge variation.

### Departure Dances and Flights

Though the sample size is small, the 2 observations of short flights preceded by short-duration waggle runs, with longer flights portended by longer dances, may contradict earlier conclusions for *A. dorsata*, *Apis mellifera* and *A. m. scutellata* [7], [23], [24] that migration dances preceding departure indicate direction but not distance. Correlation between dance duration and flight distance for the 2 observed short flights approximates earlier findings for foraging flights [25]. Of course, it may also be that dancing and departure behavior of bivouacking bees, or those selecting a nest site at the end of migration, simply differs from the behavior of bees initiating migration, i.e., departing from comb. This hypothesis is supported by the bivouacking dancers’ showing unanimity in the duration of their waggle dances in this study, compared to the great variation witnessed among dancers at the initiation of migration [7], [23], [24]. The witnessed flights and dances, short and long, indicate that migration may consist of multiple steps of variable length.

### Conservation Implications of Stopover Sites

Pollinators are in decline worldwide [2], [26]–[28]. Giant honey bees are not only a splendid spectacle but are indispensable pollinators of cultivated crops [2] and wild flowering plants [1]. They manufacture honey and other hive products, and are culturally revered in Asia [8]. Their current population decline and vulnerability to human impacts [8] and “local extinctions across extensive areas” [29] make them deserving of “the sort of conservation attention that is normally reserved for charismatic vertebrates” [1].

Though attention has been paid to disappearance of giant honey bee nesting sites in large trees [8], [30], none has been given to heretofore-unknown BCSS’s. *A. laboriosa* may be in serious decline in Nepal due to threats to wintering clusters from forest clearing and grazing [31]. Migrating *A. dorsata* need tree branches in which to rest; none of the bivouacs from this or other studies have rested elsewhere. I speculate that they have used trees on the site of the Mae Hong Son BCSS for millennia. Doubtless some tree species are more desirable than others. For example, though the BCSS was lined with tall *Eucalyptus* trees, I never saw a bivouac in this smooth-barked species, to which it may be difficult for bivouacs to cling. No clusters utilized nearby, numerous pomelo trees. Factors such as shade [18] and angle of tree branches may be important, as they are in nest site selection [32]. Plentiful nectar from flowering plants, to fuel further flight, would also be critical to bivouacking bees, as would water for evaporative cooling.

Populations of many migratory animals, and the migrations themselves, have been damaged or destroyed by loss of specific habitat crucial to vulnerable stages of the animals’ life histories [33]–[35]. Insects are not immune to such loss [36], [37]; monarch butterflies (*Danaus plexippus*) [38] and Rocky Mountain locusts (*Melanoplus spretus*) [39] provide vivid examples. With ongoing rapid destruction of Southeast Asian forests [40]–[43], BCSS’s may represent ecological or population bottlenecks [44]–[46] whose disruption could be responsible for a substantial portion of giant honey bees’ decline and/or loss of their phenomenal migrations. There is a pressing need for further study; for example, to discover other migration waypoints, and to determine the bees’ flexibility in accepting altered habitat, different tree species and alternative kinds of resting substrates in established BCSS’s.

## Methods

### Ethics Statement

Permission for access to the study site was granted by the director of the Mae Hong Son Agricultural Research and Development Center. No permits were required for very limited insect collecting. Field studies did not involve endangered or protected species.

### Survey Methods and Measurements

Commencing Sept 7, at the beginning and end of every day I surveyed the area for newly arrived and departed bivouacs, by walking or bicycling slowly for 2.5 km along the road flanking the west side of the mango orchard. This twice-daily transect included close inspection of the orchard, and of trees on both sides of the road for ≈1 km north and south. I also patrolled the site during lulls in colony activity, and sometimes found new swarms as I made rounds studying bivouacs already present. Field workers occasionally told me of swarms and their arrival times.

At least twice daily I surveyed the macadamia and pomelo orchards to the west. No bivouacs were encountered in the pomelos. I found several bivouacs in the macadamias. Approximately weekly I searched the other mango orchard, west ≈250 m from the river, but never encountered bees there. I also regularly traveled through northern Thailand both near the study area and well outside it, constantly looking for more bivouacs without success. This included, for example, a daily bicycle ride of ∼10 km to and from the village of Mae Hong Son, through human settlements, agricultural and forested land. At the height of the 2009 bivouac season I rafted the Pai River from Pai to Mae Hong Son (∼70 km), looking unsuccessfully along the shores for bivouacs.

Bivouacs were distinguishable by their widely varying sizes and shapes. Upon finding a new bivouac I attached an identifying tag to a nearby branch. With the exception of 2 that took short flights that I was able to track, bivouacs did not move around the study site before leaving it completely, so there was no potential for confusing one with another.

I recorded arrival time as the date I first saw the swarm. Often this was very precise, as I actually saw the swarm land. In rare cases that date could be late by as much as several days, if a newly arrived swarm went unnoticed. I recorded departure time as the date at which I first noted the bivouac’s absence. Again, in many cases I saw swarms depart the study site; in other instances the swarm may have left the previous afternoon but its absence was not noticed until morning. In calculating the mean length of stay, I recorded a stay as 0.5 day if it departed the same day it arrived, 1 day if its absence was noticed the day after arrival, 2 days if it was gone 2 days after its arrival, etc.

I was unsure of the precise arrival time of the first swarms seen in 2009. They were not present during a preliminary search on 1 September, but I did not survey the orchard again until 7 Sept, when I found the first bivouacs. I have thus interpolated 4 Sept as their arrival date. In 2010 I left Thailand on 19 November, when 5 bivouacs were still present, and was unable to record their departure dates. In calculating duration of stay, I used 19 November as the date of departure, so these calculations are overly conservative.

I used a tape measure to measure colony size and height at which swarms rested. For occasional lofty colonies, I estimated height by sighting against a 2-m reference pole, and colony dimensions using the known 17-mm length of an *A. dorsata* worker as a reference. Because I could not always see all sides of distant or inaccessible colonies, I did not attempt to estimate swarm volume. Rather, I recorded maximum length and width of the colonies. I present these measurements as approximate surface area of one side of the bivouac. Their depth was approximately the diameter of the branch on which they rested, normally ≈10–20 cm.

I employed unpaired t-tests to test the hypotheses that mean swarm size, mean bivouac resting height and mean length of stay varied between the 2 years.

I patrolled the swarms on a 30–60 min circuit, observing any dancing worker bees, and apparent preparations for swarm flight. Bivouacs were often low in the trees and easily viewed from 1–3 m with the unaided eye. I used Leupold™ 10×42 binoculars to view higher swarms. I photographed and video-recorded the bees’ behavior using Canon Power Shot™ S3 IS or SX10 IS digital cameras.

### Departure Dances and Flights

I measured duration of waggle dance circuits [47], [48] to the nearest second with a digital stopwatch. For bees dancing repeated circuits a mean time was calculated, again to the nearest second, though individual bees were remarkably consistent in their dances. Note that for longer dances circuit integrity breaks down; a dancing bee performs a waggle run, but instead of returning to the site where the run began, the dancer often turns and does another waggle run, or moves to another site on the mantle and does its waggle run [13], [25]. For dances lasting >3 s, I therefore measured duration just of waggle runs, not entire circuits. I estimated by eye the angle of waggle runs to the nearest 30°. For example, a dance to 12 o’clock was labeled 0°, a dance in the 1 o’clock direction was 30°, 2 o’clock was 60°, etc.

When a swarm flew, I used a compass to determine its flight direction to the nearest 5° by sighting a line from point of takeoff to point of disappearance or observed landing. I used the NOAA Sun Location Calculator [49] to find the sun’s azimuth at flight time. To test the hypothesis that dance direction and duration are correlated with flight direction and distance, I then compared the direction and, when possible, distance of the flight to that indicated by the dances [25], [47], [48], [50]. For presentation here, I have converted directions of observed dances to their indicated directions of flight, by adding the angle of dancing to the sun’s azimuth at the time [25].

### Statistical Analysis and Voucher Specimens

Statistical tests were employed following Mead *et al*. [19] and Batchelet [20]. Voucher specimens are housed in the University of Wyoming Insect Museum, Laramie, WY, USA.

## Supporting Information

Video S1 **Several bees dance 8-s waggle runs as a large bivouac prepares for a long flight out of the study area.** Note discreet waggle runs of individuals separated by a looping movement; no complete circuits are performed.
(MP4)

Video S2 Several bees dance 2-s complete circuits as a medium-sized bivouac prepares for a short flight of 150 m.
(MP4)

Video S3 **An agitated bivouac departs its resting place.** Elapsed time from liftoff until branch was vacated was approximately 60 s.
(MP4)
